# Ytterbium-doped fiber laser as pulsed source of narrowband amplified spontaneous emission

**DOI:** 10.1038/s41598-019-49695-9

**Published:** 2019-09-10

**Authors:** Pablo Muniz-Cánovas, Yuri O. Barmenkov, Alexander V. Kir’yanov, José L. Cruz, Miguel V. Andrés

**Affiliations:** 1 Centro de Investigaciones en Óptica, Loma del Bosque 115, León, Guanajuato Mexico; 2 Department of Applied Physics and Electromagnetism-ICMUV, University of Valencia, Burjassot, Spain; 3 National University of Science and Technology “MISIS”, Moscow, Russian Federation

**Keywords:** Fibre lasers, Optical physics

## Abstract

We report random noise pulsed regime of an ytterbium-doped fiber laser arranged in common Fabry-Perot configuration. We show that the laser output obeys the photon statistics inherent to narrowband amplified spontaneous emission and that the noise pulsing is properly addressed in terms of probability density and autocorrelation functions. Our novel approach reveals, in particular, that the regime’s coherence time dramatically shortens, from few ns to tens ps, with increasing laser power.

## Introduction

Fiber lasers (FLs) are very attractive devices that found a great number of commercial applications. These light sources are adopted for fully fiberized optical schemes with no any free-space components and demonstrate very high optical and electrical to optical conversion efficiency. FLs operate in a variety of regimes, characterized by narrow (from a tens Hz)^1^ to a very large optical band^2^, in continuous-wave (CW)^3^ and pulsed regimes including Q-switched, mode-locking and soliton operations where pulse width is ranged from hundreds of nanoseconds down to tens of femtoseconds^4–7^. Furthermore, modern optical materials e.g. low-dimensional structures, rapidly emerging as new types of nanoscale photonic and optoelectronic components, have been recently applied as fast saturable absorbers in mode-locked and soliton fiber lasers^8–10^.

CW double-clad (DC) ytterbium-doped fiber lasers (YDFLs) are featured by excellent power budget, mostly due to the absence of excited state absorption in the system of Yb^3+^ ions. Such lasers when pumped at ~975 nm demonstrate optical efficiency of up to ~80–83%^11,12^, limited by Stokes-shift loss and, sometimes but not necessarily, by the concentration^13,14^ and photodarkening^15–17^ phenomena. Another effect that may deteriorate YDFL efficiency is specific broadening of the laser line when YDFL cavity is formed by fiber Bragg grating (FBG) couplers^18–20^; the broadening is usually ascribed to spatial hole burning and self-phase modulation (SPM) produced by fluctuating intensity^18^ and leads to partial leakage of laser power on a highly reflective (HR) FBG or to spectral “rounding” of a lowly reflecting (LR) FBG, respectively^20^.

Output power of DC YDFLs is scalable from several watts to tens kW^11,12,21,22^ or few hundreds kW^22–25^ at single-mode and multimode operation, respectively. When cavity Q-factor of YDFL is low and thus YDF gain is high, the laser turns to the regime of random kW pulses (rogue waves), driven by stimulated Brillouin scattering^26–28^.

FL intensity noise originates from intensity fluctuations of amplified spontaneous emission (ASE) that is added to the laser wave at its each round-trip along the laser cavity; intuitively, the lower reflectivity of the output cavity coupler, the stronger impact of ASE noise on the laser output signal. It is known that when FL operates in a pulsed regime the noise results in timing jitter of the laser pulses^29–31^. As noted in^18^, in the case of CW YDFL, high-intensity noise may boost quasi-CW oscillation through longitudinal modes dephasing^20^. FL noise should play an important role in many applications, especially of high-power FLs where nonlinear effects are significant; thus, a detailed study of this phenomenon is worth of attention.

Herein we report a striking effect of excessive intensity noise in a CW DC YDFL with moderate output (up to 22 W), assembled in common Fabry-Perot cavity geometry with two FBGs used as narrowband reflectors. We show that the laser operates in the regime of noise pulses with random magnitudes and widths, not in true CW or quasi-CW regimes as one would expect. We also show that the laser photon statistics depends on laser power (and hence on laser linewidth) and that its behavior is similar to that of narrowband ASE; some high-amplitude noise events occurring with low probability reach powers more than an order greater than the mean laser power. We further discuss the results of a statistical analysis of laser signal, specified for both polarized and unpolarized outputs, which permits one to understand better the physics behind the excessive intensity FL noise. The spectral and coherence properties of the laser are addressed in detail.

The paper is organized as follows.

First, we demonstrate that the laser spectrum’s width increases with increasing output power and that the rate of the process at lower output powers is lower than at higher ones, given that in the latter case the nonlinear fiber length is significantly less than the cavity one. These data are necessary for addressing the statistical pattern of laser photon noise.

Second, we treat the YDFL’s noise pulsing in terms of autocorrelation function for intensity. It is shown that the coherence time of lasing, referred to as the zero-order peak of this function, steadily decays, from ~1 ns to <100 ps, with increasing laser output power from 1 W to 22 W. Furthermore, it is demonstrated that at low-power operation the laser retains the noise temporal pattern, established during preceding round-trip (RT) interval, for a large number of following RTs and with a high degree of correlation; at high-power operation, the laser does not demonstrate such type of correlation.

Third, we reveal that, for output powers varying in a broad range, the laser does not operate neither in true CW nor quasi-CW mode but instead oscillates noise pulses and that this emission can be suitably described by probability distribution function (PDF), congruent to that used for narrowband amplified ASE^32^. Note that such type of noise pulsing was recently reported for an actively Q-switched Erbium-doped fiber laser and conversion of its output for supercontinuum (SC) generation^33,34^. Since CW or gain-switched CW YDFLs with Fabry-Perot cavity are now-a-days used for SC generation, too^35–38^, the knowledge about noise features of such lasers becomes crucial.

## Experimental Setup and Basic Laser Characteristics

**Figure 1:**
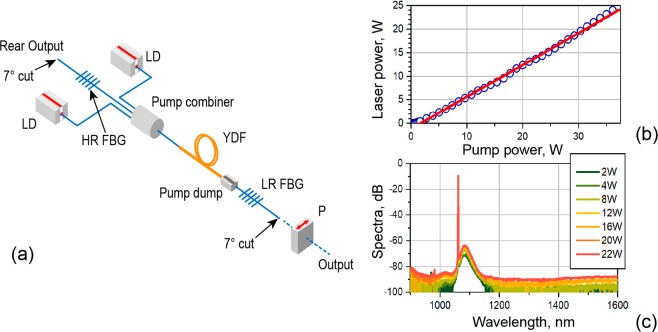
(**a**) Experimental arrangement of YDFL. (**b**) Laser output *vs*. pump power. Circles are experimental points and solid line is a linear fit. (**c**) Laser broad-band optical spectra measured at different laser powers (specified in inset).

In Fig. 1(a), we present an experimental setup of the YDFL, assembled in Fabry-Perot cavity with two home-made narrowband 1061-nm FBG-couplers with reflection coefficients of ~100% (HR-FBG) and 10% (LR-FBG), respectively. The YDF used was a standard DC fiber (Nufern, SM-YDF-5/130-VIII) with cladding absorption of ~1.65 dB/m at 975 nm and mode field diameter (MFD) of 6.5 μm at the laser wavelength (1061 nm). YDF length was 15 m that corresponds to ~25-dB pump absorption; the cavity length, *L*_*c*_, was 19 m, which corresponds to RT interval of 188.35 ns. YDF was pumped by two commercial 976-nm laser diodes (LDs) through a (2 + 1) × 1 pump combiner. A fiber polarizer (P) was added to the scheme for studying statistical properties of polarized light (it was removed when the case of unpolarized light was explored).

Laser signal was detected by a 25-GHz InGaAs Schottky photodetector (Newport, model 1414), connected to a 16-GHz real-time oscilloscope (Tektronix, DPO71604C). The cumulative 3-dB RF band (*B*_*el*_) of this set of equipment extended from DC to ~15.5 GHz, enabling detection of light pulses as short as ~30 ps. At measurements, laser power was always attenuated to ~1 mW to ensure the photodetector’s operation well below saturation.

Laser slope efficiency and threshold pump power were measured to be ~70% and ~2 W, respectively (Fig. 1(b)). The YDFL’s optical spectra were recorded with an optical spectrum analyzer (OSA) (*Yokogawa*, AQ6370D). The spectra (Fig. 1(c)) reveal a narrow laser line that exceeds the broadband Yb^3+^ ASE by 55–60 dB; no Raman-scattering was observed in the spectrum even at the highest laser power.

## Results and Discussion

**Figure 2:**
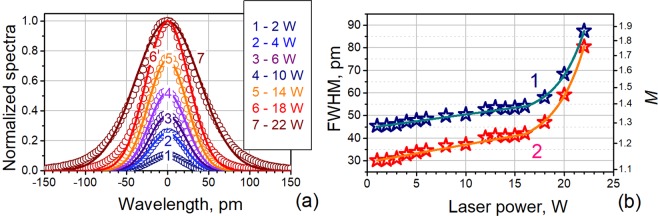
(**a**) YDFL spectra measured at different laser powers (specified in inset) in function of detuning from the laser peak wavelength. All spectra are normalized to the spectral maximum at 22 W of laser power. Circles: experimental data; lines: Gaussian fits. (**b**) FWHM bandwidths of YDFL spectra for different laser powers: symbols are experimental data and lines are fits; lines 1 and 2 stand for FWHM values, measured directly with OSA and after deconvolution, correspondingly. The right scale shows the number *M* of independent states of ASE for polarized light (see the discussion below).

The narrow-band laser spectra measured with 34-pm OSA resolution in the whole range of laser powers, are shown in Fig. 2(a). As seen, the spectra monotonously broaden with increasing laser power, the effect observed in powerful (tens watts and higher) CW YDFLs. All spectra are fit by the Gaussian function with the residual sum of squares *R*^2^ > 0.996. The spectra’ full widths at half maximum (FWHM) in Gaussian approximation *vs*. output power are plotted by blue asterisks in Fig. 2(b); curve 1 fits the data. In turn, “real” laser bandwidths, found by deconvoluting the spectra with OSA’s Gaussian-like response to a narrow-line (<200 kHz) probe signal, are built in the figure by red asterisks and fit by curve 2. Note that *M*-values on the right scale of Fig. 2(b) (to be defined further) found from FWHM data are required for discussing laser statistical properties; see below.

As seen from Fig. 2(b), the slope of the lines is by more than one order less for lower laser powers (up to *P* ~18 W) than that for higher ones. At *P* ~ 18 W, above which the slope dramatically changes, the nonlinear fiber length *L*_*NL*_ found as (*γP*)^−1^ ^39^ is 7 m, i.e. by ~2.5 times less than the cavity length (here *γ* = *n*_2_*ω*/(*cA*_*eff*_) is the nonlinear parameter, *n*_2_ = 4.3 × 10^−20^ m^2^/W^40^ is the nonlinear refractive index of fused silica at 1.06 μm, *ω* is the light angular frequency, and *A*_*eff*_ = π(MFD/2)^2^ = 34 μm^2^ is the effective laser beam area in the fiber). Given that intracavity radiation reaches maximum power in an YDF section adjacent to the laser output (refer to Figure 4 in ref.^41^ where a similar YDFL is simulated using a contra-propagated waves’ model), the nonlinear effects should be most prominent in this section, too.

**Figure 3:**
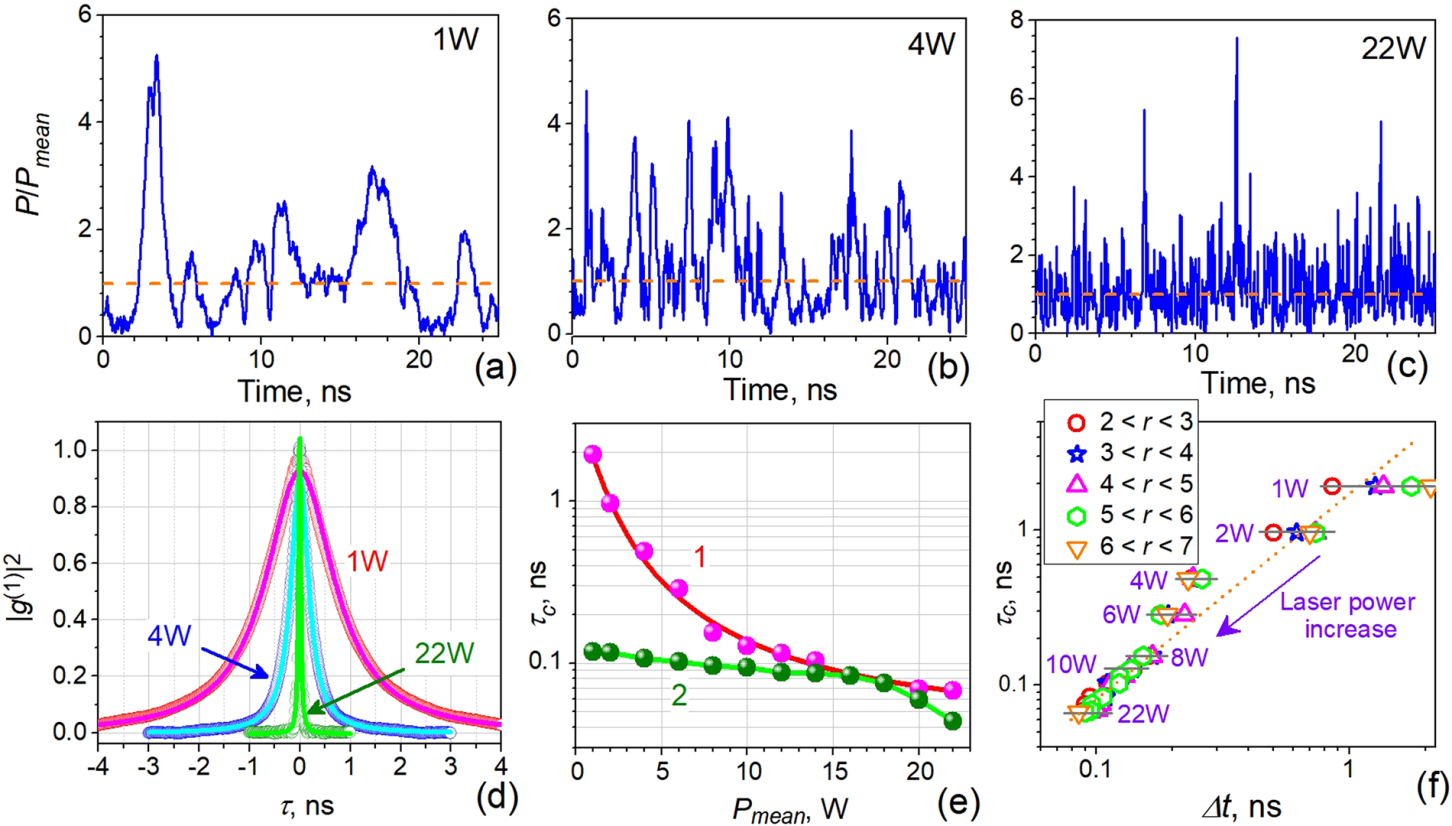
(**a**–**c**) Oscilloscope traces of YDFL noise pulses for three different laser powers (indicated in each plot). The horizontal dash lines indicate *P*_*mean*_-values of the signals. (**d**) Zero-order peaks of the squared first-order coherence for the same laser powers. (**e**) *τ*_*c*_ versus *P*_*mean*_. (**f**) Relation between °*τ*_*c*_ found from curve 1 in (**e**) and the most probable value of noise pulses’ width *Δt*. Symbols represent the data for different intervals of noise pulse magnitude (specified in inset). The violet arrow shows the direction in which the laser power increases (some values of laser power are marked near experimental points). The dot line is linear fit in log-log plot. Inset shows the ranges of ratio *r* = *P*_*peak*_/*P*_*mean*_ (*P*_*peak*_ is the peak power of noise pulses).

Just above threshold, the laser operates in the regime of random (noise-like) relaxation oscillations, the effect commonly observed in the most of CW lasers^42^. As pump power increases, the laser continues to operate in this regime of noise pulses with random magnitudes, widths, and intervals of sequence, with some of them overlapping; see Fig. 3(a–c) where 25-ns snapshots of the laser signal are exemplified for three values of the mean laser power, *P*_*mean*_, differing by ~6–7 dB: 1 W (a), 4 W (b), and 22 W (c). It is seen that with *P*_*mean*_ growing the density of intensity fluctuations steadily increases while the average width of noise pulses simultaneously decreases.

Another important parameter of this kind of lasing is the coherence time, *τ*_*c*_. In order to analyze the laser’s coherent properties in terms of *τ*_*c*_, we applied the formalism of autocorrelation function for intensity, which is interrelated with the second-order coherence, *g*^(2)^(τ)^43,44^ as follows:

1 $${g}^{(2)}(\tau )=\frac{I(t)I(t+\tau )}{I{(t)}^{2}}=\frac{P(t)P(t+\tau )}{{({P}_{mean})}^{2}}$$

where *I*(*t*) is the signal intensity, *P*(*t*) is the laser power and *τ* is the shift with respect to current time *t*. Accordingly, the first-order coherence, *g*^(1)^(*τ*), is found from *g*^(2)^(*τ*) as^45^:

2 $${|{g}^{(1)}(\tau )|}^{2}={g}^{(2)}(\tau )-1$$

Three examples of the zero-order peaks of |*g*^(1)^(*τ*)|^2^ calculated for YDFL powers 1 W, 4 W, and 22 W are shown in Fig. 3(d). As seen, the experimental data (circles) are precisely fit by the Lorentzian function (solid lines) that models collisionally-broadened thermal light^44^. Note that the averaging time in calculations was 312.5 μs, given by the oscilloscope’s limit of 31.25 mega-samples. In turn, the dependences of *τ*_*c*_ found from these fits *vs*. laser power are plotted in Fig. 3(e), where curve 1 stands for *τ*_*c*_ found from |*g*^(1)^(*τ*)|^2^ and curve 2 for the value obtained from the deconvoluted width of the optical spectra (refer to line 2 in Fig. 2(b)). The width of the autocorrelation function steadily decays, from ~1 ns to tens picoseconds, with increasing laser power from 1 W to 22 W.

Another important observation is that the autocorrelation function’s width (and so *τ*_*c*_, too) is close to the mean width of noise pulses (see ref.^43^); this statement was experimentally verified for the laser under study by comparing these two variables shown in Fig. 3(f) in log-log scale. The noise pulse widths were measured for different ranges of the pulse magnitudes (the technique is described in ref.^32^) as the most probable values found from corresponding PDFs for counts of the noise pulses. It is seen that if laser power is low, pulse width increases synchronously with pulse magnitude (see the data for 1 W and 2 W), whereas at higher powers (above 2 W), pulse width gets scattered but slightly, by ±10% or less. Hence, we may conclude that the laser coherence time (found as the width of intensity autocorrelation function) is nearly equal to the width of ASE noise pulses and that both quantities decrease with increasing laser power.

**Figure 4:**
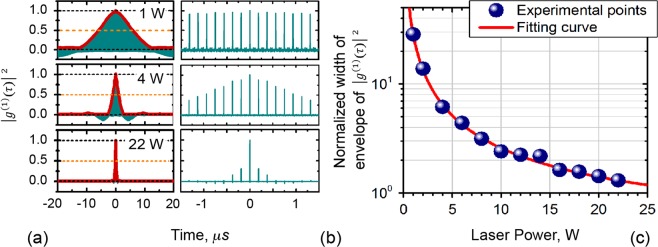
(**a,b**) Normalized autocorrelation function |*g*^(1)^|^2^ obtained for three laser powers (1, 4, and 22 W). (**c**) Dependence of |*g*^(1)^|^2^ envelope’s FWHM normalized to RT interval (*ξ*) *vs*. laser power. Circles show the experimental data and solid line is the fit.

Special attention was paid to study of the behavior of autocorrelation function in a broad temporal range. Figure 4(a) uncovers the trends of |*g*^(1)^(*τ*)|^2^ within the temporal interval from −20 μs to 20 μs. The green areas under the red envelopes are filled with trains of peaks, separated by RT interval (*T*_*RT*_) (the trains are zoomed in Fig. 4(b)). These areas correspond to partially overlapping noise pulses at *t*_*k*_ = *kT*_*RT*_, where *k* is an integer (the zero-order peak (*k* = 0) was already discussed above). Note that in gaps between the peaks the first-order coherence is nearly zero. Regarding the higher-order peaks of the autocorrelation function (herein, “lobes”), their magnitudes are seen to drop with increasing laser power. This tendency, for the whole range of laser powers, is highlighted by Fig. 4(c) where the results of calculating FWHM of the lobes’ envelope after normalization by *T*_*RT*_ are shown by the blue symbols (the red line is the best fit by the generalized logistic function); the vertical axis in the plot is scaled in *T*_*RT*_-numbers *ξ* at which the envelope’s width decays twice. As seen, at the lowest power (1 W) FWHM is approximately thirty *T*_*RT*_ (*ξ* ≈ 30) whereas at the highest power (22 W) it is reduced to one *T*_*RT*_ (*ξ* ~ 1), thus indicating that the pattern of laser noise, established during one RT, does not correlate with those arisen during the other RTs.

Note here that similar behavior of the intensity autocorrelation function (*viz*. visibility of the interference pattern) was reported for a He-Ne laser^46^, where coherence gets enhanced each time when the difference between pathlengths in two interferometer arms is equal to the integer number *k* of RT cavity length (2*kL*_*c*_). In the case of YDFL under study this effect exists at the lowest laser powers (1 W to 4 W) only.

**Figure 5:**
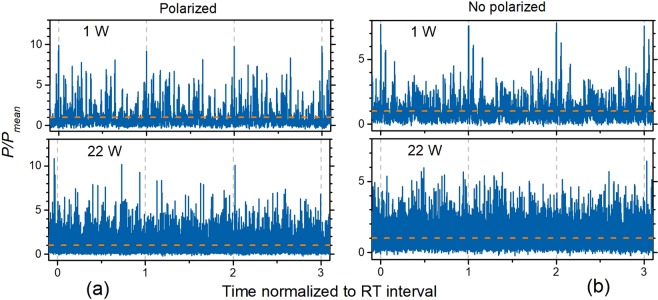
Oscilloscope traces captured for (**a**) polarized and (**b**) unpolarized YDFL output. The horizontal dash lines mark the mean levels of laser power; the vertical dashed lines delimit the RT intervals.

The effect of strong correlation of noise pulses arising in neighboring RT intervals at low laser power is illustrated by Fig. 5, where the examples of laser signal captured during ~3 RTs for *P*_*mean*_ = 1 W (upper traces) are snapshotted. The left and right plots demonstrate the snapshots acquired for polarized and unpolarized light, respectively. As seen, the pattern of noise pulses in consecutive RT intervals is almost the same (compare the areas delimited by the vertical dash lines); slight discrepancy between the patterns within the adjacent RTs appears because of the photodetector noise and the oscilloscope’s digitalization. In other words, the laser in this case retains the pattern of noise pulsing, established in the cavity during one RT, for many (~30; see Fig. 4(c)) subsequent RTs. At the high laser power, *P*_*mean*_ = 22 W (see the two lower snapshots in Fig. 5), noise pulses do not demonstrate any periodicity in subsequent RT intervals; hence, in this case laser noise is fully random and the high-order lobes of coherence rapidly fade (refer to the lower plot in Fig. 4(b)).

Let us examine whether YDFL noise pulses obey the statistics of narrow-band ASE^32,47,48^. This type of statistics is characterized by PDF that depends on number *M* of independent states of narrow-band ASE inside laser cavity. For the Gaussian optical spectrum (refer to the right scale in Fig. 2(b)), *M*-number is found as^47,48^:

3 $$M=s\frac{\pi {({B}_{opt}/{B}_{el})}^{2}}{\pi ({B}_{opt}/{B}_{el}){\rm{erf}}\,[\sqrt{\pi }({B}_{opt}/{B}_{el})]-[1-\exp (-\pi {({B}_{opt}/{B}_{el})}^{2})]}$$

where *B*_*opt*_ is the optical bandwidth in terms of the deconvoluted spectral width (line 2 in Fig. 2(b)) and *s* is the number of polarization states (1 or 2, for polarized or unpolarized light, respectively). Note that the extreme cases of *M* = 1 (polarized light) and *M* = 2 (unpolarized light) may be only considered if optical spectrum is very narrow (*B*_*opt*_/*B*_*el*_ ≪1).

**Figure 6:**
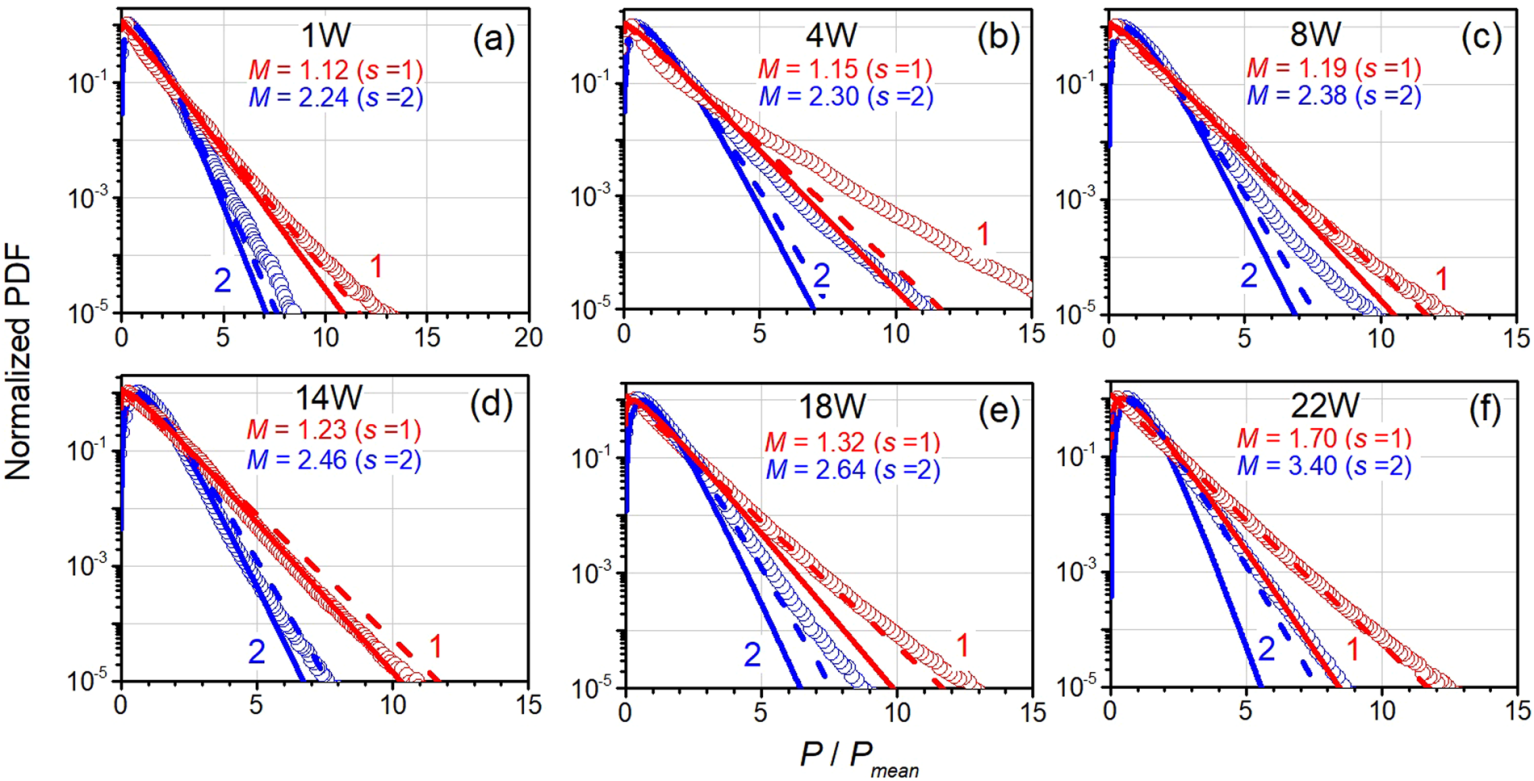
Normalized PDFs of photodetector signals obtained for polarized light (red lines and symbols, marked as (1) and unpolarized light (blue lines and symbols, marked as (2)). Symbols are experimental points, lines are PDFs calculated using Eq. (4) for *M* found from the optical spectra (solid lines) and for the extreme case of *M* = 1 (polarized light) and *M* = 2 (unpolarized light) (dash lines). Laser power is indicated in each plot.

The PDFs obtained for a set of laser powers are exemplified in Fig. 6 by symbols, with curves 1 and 2 standing for polarized (*s* = 1) and unpolarized (*s* = 2) light, respectively. *M*-values for polarized light were found using Eq. 3 and FWHMs of the laser spectra (refer to line 2 in Fig. 2(b)) being 1.12, 1.15, 1.19, 1.23, 1.32, and 1.70; for unpolarized light *M*-values are two times larger (*M*-values are indicated in each plot). The fits (solid lines in Fig. 6) for the experimental PDFs were found using the *M*-fold degenerate Bose-Einstein distribution, valid in the approximation of narrow-band ASE^47,48^:

4 $$P(n,\bar{n},M)=\frac{(n+M-1)!}{n!(M-1!}\frac{{(\bar{n})}^{n}}{{(1+\bar{n})}^{n+M}}$$

where $P(n,\bar{n},M)$ is the probability of counting *n* ASE photons by photodetector during average time *T* = 1/*B*_*el*_ and $\bar{n}$ is the mean photon count. Note that *n* and $\bar{n}$ are proportional to instantaneous laser power, *P*(*t*), and mean laser power, *P*_*mean*_, respectively.

As seen from Fig. 6, the best agreement between the experimental PDFs and the fits is attained for *P* = 14 W (plot (d)), indicating that the laser operates in regime of narrow-band ASE (“thermal light”). However, at lower and higher powers, the simulated PDFs deviate from the experimental ones. For instance, for *P* varying between 2 W and 8 W, the tails of the experimental PDFs lie above their theoretical counterparts, whenever found for the extreme cases *M* = 1 (polarized light) or *M* = 2 (unpolarized light), whereas for *P* ≥ 18 W the modeled and the experimental PDFs merge.

We can infer that the high-power events (to be tentatively associated with rogue waves or their seeds^49–52^) arise in the experiment more frequently than it is predicted by the modeling (Eqs 3 and 4). Moreover, at high laser powers, the photon statistics are suitably approximated by PDFs built for *M* = 1 (polarized light) (that holds in the wave turbulence theory for weakly nonlinear regimes^53–55^) or for *M* = 2 (unpolarized light), in spite of the optical spectra are not narrow enough to obey the relation *B*_*opt*_/*B*_*el*_ ≪1.

Let us make insight to the discrepancies between the experimental PDFs and their fits. Note that at relatively low laser powers the effective length of nonlinear interaction of the laser wave with the fiber *L*_*eff*_ is long as the noise pattern is preserved in this case for many RT intervals (*ξ* ≫1); see Fig. 4(c). Particularly, at very low laser power (1 W) the discrepancy is quite small because of too low magnitudes of noise pulses (Fig. 6(a)). At higher laser powers (from ~2 W to ~10 W), the discrepancy is growing because although *L*_*eff*_ is still large the magnitudes of noise pulses become greater. At further increasing laser power (from 12 W to 16 W), *L*_*eff*_ gets shorten and thus starting to affect the optical spectrum’s width (see Fig. 4); accordingly, the histograms of laser noise become precisely fit by the Bose-Einstein distribution with *M*-value either found from the optical spectrum or from *τ*_*c*_; refer to Fig. 6(d). However, at the highest laser powers (≥18 W), *L*_*eff*_ is very short (*ξ* ≤ 1) and the laser field (in the form of highly energetic noise pulses) begins to interact with the gain fiber too strong that the theory applied here fails to provide agreement between the experimental and modeled PDFs (see Fig. 6(e,f)).

Finally, we ought to note that any laser inherently oscillating in regime of noise pulses may be classified as a CW noise laser, likewise mode-locked or soliton lasers that operate in noise-like pulsing^56–59^. The clue difference between the noise parameters in these two types of laser is that for the latter the autocorrelation function (and so the mean width of noise pulses) is measured by fractions of picoseconds whereas for the former these are of the order of tens picoseconds.

## Conclusion

In this paper, we presented a study of the noise features of a double-clad ytterbium-doped fiber laser (YDFL) with Fabry-Perot cavity formed by two fiber Bragg gratings. We revealed the following basic results:

(i) The YDFL operates in the regime of noise pulses with random magnitudes and widths, on the contrary to what one would expect (conventional continuous, or quasi-continuous, wave oscillation); width of noise pulses steadily drops with increasing laser power.
(ii) The autocorrelation function for long traces of the laser signal presents a series of peaks, separated by one round trip (RT) interval of light in the cavity. The laser coherence time, interrelated with the zero-order peak autocorrelation function, notably decreases, from ~1 ns to tens picoseconds, with increasing laser power from 1 W to 22 W; its value is close to the most probable width of noise pulses.
(iii) The presence of lateral peaks (“lobes”) in the intensity autocorrelation function reveals that the laser retains the pattern of noise pulsing, established during one RT, for a considerable number of consequent RTs; however, this effect depends on laser power: the “memory” of noise pattern fades from ~30 RT at low powers to ~1 RT or less at high powers, indicating transition of the YDFL from partly coherent to fully incoherent regime.
(iv) The histograms of photon counts, or probability density functions (PDFs), are described by the *M*-fold degenerate Bose-Einstein distribution with *M*-value dependent on laser power: at low (~1 W) and medium (~14 W) laser powers *M*-values correspond to the optical spectrum width, thus confirming that the YDFL operates in the regime statistically equal to narrow-band amplified spontaneous emission. At higher laser powers (>18 W), probability of high-power events is significantly higher than the expected one, so that the experimental photon statistics match PDFs characterizing the extreme case of very narrow optical spectrum (*B*_*opt*_/*B*_*el*_ ≪1) with *M*-values being the smallest allowable (*M* = 1 and *M* = 2 for polarized and unpolarized light, respectively). Within a limited range of laser powers, from 2 W to 8 W, the photon statistics has too long tails to be correctly described by Bose-Einstein distribution; this happens due to retaining of the shape of noise pulses in train over many RT intervals and results in long effective distance of nonlinear interaction of the laser wave with the optical fiber.

## Data Availability

The data generated and/or analyzed during the current study are available from the corresponding author upon request.
